# From genotype to microbiome function: a gene–metabolite–microbiome pathway shaping plant nutrient acquisition

**DOI:** 10.1111/nph.71457

**Published:** 2026-07-24

**Authors:** Zhiqiang Pang, Peng Yu

**Affiliations:** ^1^ Plant Genetics, TUM School of Life Sciences Technical University of Munich Freising 85354 Germany; ^2^ CAS Key Laboratory of Tropical Plant Resources and Sustainable Use, Xishuangbanna Tropical Botanical Garden Chinese Academy of Sciences Mengla 666303 China; ^3^ Emmy Noether Group Root Functional Biology Institute of Crop Science and Resource Conservation (INRES), University of Bonn Bonn 53117 Germany

**Keywords:** functional microbiome, gene–metabolite–microbiome axis, microbial recruitment, plant microbiome, root exudates

## Abstract

This article is a Commentary on Xu *et al*. (2026), **252**: 817–828.

Over the past decade, plant microbiome research has advanced rapidly, revealing that host genetics and root exudates play central roles in structuring microbial communities. Yet the mechanistic puzzle remains unresolved. Root exudates, comprising sugars, amino acids and organic acids, are broadly utilizable carbon sources, theoretically capable of supporting a wide spectrum of soil microbes (Pang & Xu, 2024; Tsai *et al*., 2025). How, then, do plants achieve the precise enrichment of specific functional guilds under defined stress conditions? How can chemically diverse compounds give rise to specific biological outcomes? In an article published in this issue of *New Phytologist*, Xu *et al*. (2026; pp. 817–828) provide an answer to this question. Employing a classic reverse genetics strategy, they systematically dissected the complete causal chain from genetic variation to microbial recruitment and ultimately to soil organic phosphorus mineralization by comparing wild‐type rice with loss‐of‐function mutants of *OsA1*, a gene encoding the plasma membrane H^+^‐ATPase. This represents a compelling example of validating plant–microbiome functional interactions at a single–gene resolution.

*Notably*, Bacillus cereus *strain FU2 carries* phoC *genes encoding acid phosphatases that mineralize organic phosphorus – a major soil phosphorus pool that can account for up to 50% of total phosphorus yet is largely inaccessible to plants*.

While it is well established that plant genotypes influence microbiome composition and function (Hu *et al*., 2018; Yu *et al*., 2021; He *et al*., 2024), the intermediate processes linking genetic variation to metabolite production, and ultimately to microbial function, remain incompletely understood. This gap has become a central bottleneck for the emerging vision of ‘designer microbiomes’, which seek to harness plant genetic control to engineer rhizosphere communities for improved nutrient acquisition and stress tolerance (Sasse *et al*., 2018; Fan *et al*., 2025; Ge & Wang, 2025).

To address this challenge, a useful conceptual framework is to consider plant genetic determinants that shape the assembly and function of associated microbial communities. Recent studies have identified candidate plant genes involved in root exudation, nutrient transport and immune signaling that influence microbial recruitment and community composition (Brachi *et al*., 2022; Su *et al*., 2024, 2025). However, most of these studies remain correlative, and the full causal chain from genetic variation to metabolite release, microbial enrichment and functional outcomes is still largely unresolved.

Against this backdrop, Xu *et al*. offer a clear mechanistic resolution to this problem. Under low‐phosphorus conditions, the plasma membrane H^+^‐ATPase *OsA1* stimulates malate exudation from rice roots, likely via membrane potential–dependent activation of anion transporters such as *OsALMT4* (Fig. 1). Although malate is a common organic acid, its elevated flux functions as a quantitative filter that preferentially enriches *Bacillus cereus*. Notably, *B. cereus* strain FU2 carries *phoC* genes encoding acid phosphatases that mineralize organic phosphorus – a major soil phosphorus pool that can account for up to 50% of total phosphorus yet is largely inaccessible to plants. Through inoculation experiments, the authors further demonstrate that this single strain can rescue the phosphorus uptake defect of *osa1* mutants, thereby closing the causal loop from genotype to microbiome assembly and function via metabolites.

**Fig. 1:**
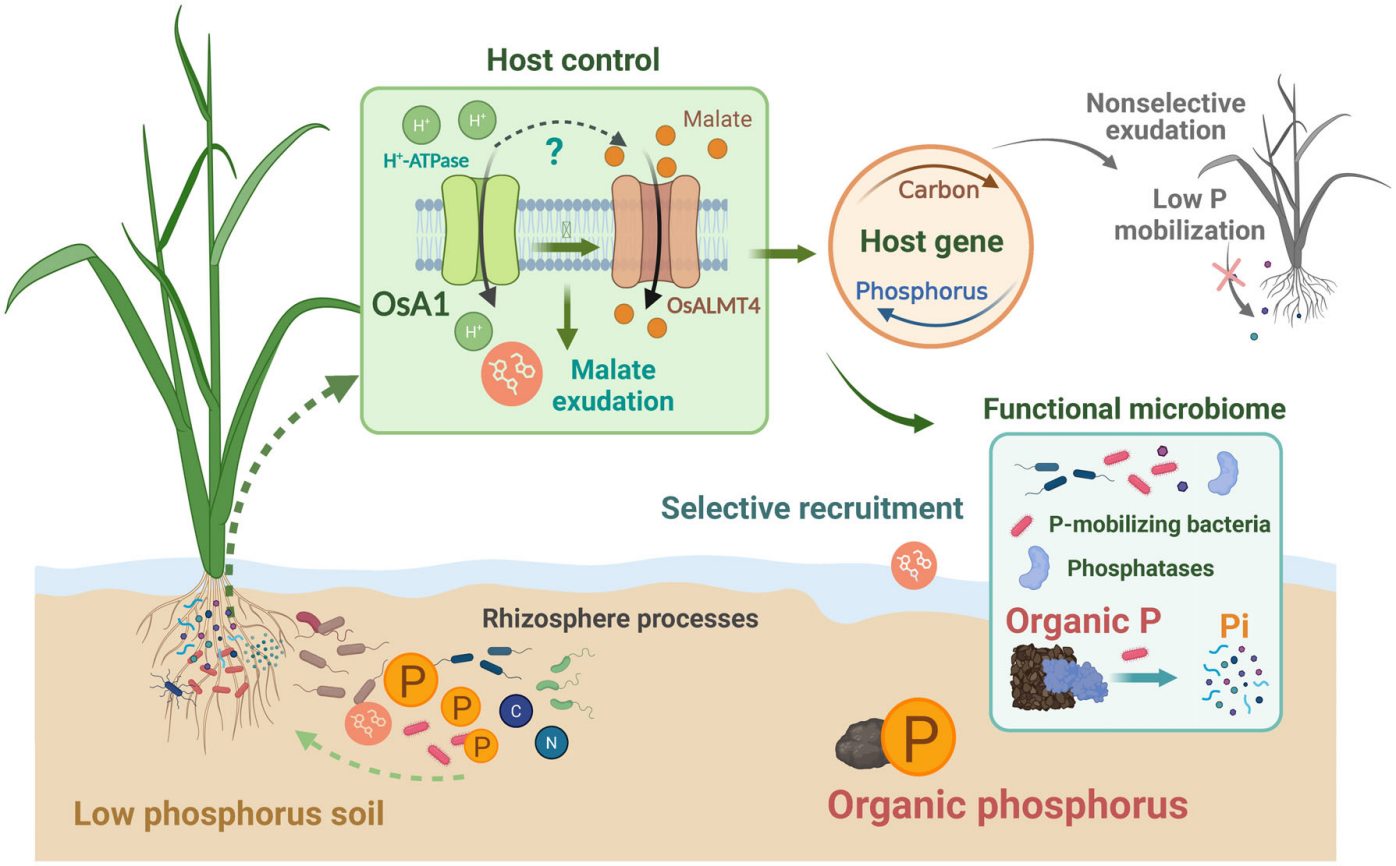
Mechanistic framework for host genetic regulation of rhizosphere microbiome assembly under low phosphorus conditions. Under phosphorus‐deficient conditions, the plasma membrane H^+^‐ATPase *OsA1* facilitates malate efflux, potentially mediated by the anion channel *OsALMT4*. The increased malate exudation acts as a selective carbon source that promotes the enrichment of specific functional microbial groups in the rhizosphere. These recruited microorganisms, including phosphorus‐mobilizing bacteria, express phosphatases that convert organic phosphorus into plant‐available inorganic phosphate (Pi). This model illustrates a gene–metabolite–microbiome–function cascade, highlighting how host genetic regulation enables precise microbial recruitment and enhances phosphorus acquisition under nutrient limitation. Some components of this figure were created in BioRender (https://BioRender.com/hyfqbza).

Beyond serving as carbon sources, root‐derived metabolites may also function as signaling molecules that shape microbial assembly and function (Tsai *et al*., 2025). Quantitative variation in metabolite flux may therefore encode ecological information that influences microbial chemotaxis, colonization and function, providing a key mechanism for specificity in microbiome recruitment. Rhizosphere microbial communities emerge from the interplay between host traits, abiotic factors, soil microbial pools and ecological processes such as competition and niche partitioning, with plant‐derived metabolites acting primarily as selective filters on an existing microbial reservoir. Plant metabolites can filter microbial communities differently because microbial taxa vary in their metabolic capacities, substrate preferences and stress tolerance traits, while abiotic conditions influence metabolite diffusion and accessibility. For example, a high‐throughput transposon sequencing study revealed that most bacterial genes required for plant colonization are substrate‐dependent, with only a small core set shared across conditions (Torres *et al*., 2025). Notably, root exudates alone could not explain these differences, highlighting the role of abiotic constraints such as porosity and aeration in determining microbial fitness.

## Microbiome‐related plant genes operate through coordinated regulatory systems

Microbiome assembly is not strictly ‘hard‐wired’ at the level of species identity but emerges from ecological processes shaped by the interplay between host regulation and environmental context. In particular, different plant genes may give rise to distinct ‘selection regimes’, enabling plants to dynamically assemble microbiomes adapted to environmental conditions (Zhang *et al*., 2019; Su *et al*., 2025; Ma *et al*., 2026). Moving forward, a key challenge will be to define the genetic architecture underlying these processes. This will require shifting from gene‐by‐gene characterization towards identifying networks of genes that collectively regulate metabolite production, microbial recruitment and ecosystem function. Such an approach may ultimately enable predictive and programmable control of plant‐associated microbiomes.

More broadly, plant genes associated with microbiome assembly and function may be better understood not as isolated loci, but as components of coordinated regulatory modules that jointly shape microbiome assembly and host environmental adaptation. Rather than selecting specific microbial taxa, plants may encode rules of engagement, genetically controlled traits such as exudation profiles and immune thresholds that bias the recruitment of functional microbial groups. An important but largely unexplored dimension is the evolutionary and ecological plasticity of microbiome‐related host genes, particularly in how their effects vary across environments (Pang & Xu, 2024; Ma *et al*., 2026). This raises the possibility that plant genomes encode flexible strategies for microbiome assembly, enabling dynamic responses to fluctuating nutrient availability and stress conditions (Fitzpatrick *et al*., 2026). Understanding how these genes evolve and are maintained across plant lineages will be critical for assessing their potential in microbiome engineering. The gene–metabolite–microbe–function cascade suggests that plant genomes may encode sophisticated strategies for microbial management.

Furthermore, the study raises important questions. To what extent do these effects depend on specific taxa such as *Bacillus*, vs broader functional redundancy within microbial communities? How stable are these interactions across diverse soils and environmental conditions? And what are the carbon costs associated with sustained exudation, and under which conditions do these investments yield net benefits? A central challenge moving forward is to reconcile single‐strain mechanisms with community‐level dynamics. While studies such as Xu *et al*. provide clear causal links between host genes and specific microbial taxa, natural microbiomes are complex and often exhibit functional redundancy. It remains unclear whether the recruitment patterns are conserved across diverse soil environments where multiple taxa compete for the same resources. Bridging this gap will require integrating reductionist approaches with community ecology to determine how gene‐mediated metabolite fluxes scale to complex microbiome assemblages. As more mechanistic blueprints emerge, the vision of designer microbiomes comes into sharper focus. The path from genotype to microbiome function is gradually becoming predictable, and potentially programmable.

## Disclaimer

The New Phytologist Foundation remains neutral with regard to jurisdictional claims in maps and in any institutional affiliations.
